# The Bacterium Endosymbiont of *Crithidia deanei* Undergoes Coordinated Division with the Host Cell Nucleus

**DOI:** 10.1371/journal.pone.0012415

**Published:** 2010-08-26

**Authors:** Maria Cristina Machado Motta, Carolina Moura Costa Catta-Preta, Sergio Schenkman, Allan Cezar de Azevedo Martins, Kildare Miranda, Wanderley de Souza, Maria Carolina Elias

**Affiliations:** 1 Laboratório de Ultraestrutura Celular Hertha Meyer, Instituto de Biofísica Carlos Chagas Filho, Universidade Federal do Rio de Janeiro, Rio de Janeiro, Rio de Janeiro, Brazil; 2 Instituto Nacional de Ciência e Tecnologia em Biologia Estrutural e Bioimagens, Rio de Janeiro, Rio de Janeiro, Brazil; 3 Departamento de Microbiologia, Imunologia e Parasitologia, Universidade Federal de São Paulo, São Paulo, São Paulo, Brazil; 4 Instituto Nacional de Metrologia, Normalização e Qualidade Industrial - Inmetro, Rio de Janeiro, Rio de Janeiro, Brazil; 5 Laboratório de Parasitologia, Instituto Butantan, São Paulo, São Paulo, Brazil; Duke University Medical Center, United States of America

## Abstract

In trypanosomatids, cell division involves morphological changes and requires coordinated replication and segregation of the nucleus, kinetoplast and flagellum. In endosymbiont-containing trypanosomatids, like *Crithidia deanei*, this process is more complex, as each daughter cell contains only a single symbiotic bacterium, indicating that the prokaryote must replicate synchronically with the host protozoan. In this study, we used light and electron microscopy combined with three-dimensional reconstruction approaches to observe the endosymbiont shape and division during *C. deanei* cell cycle. We found that the bacterium replicates before the basal body and kinetoplast segregations and that the nucleus is the last organelle to divide, before cytokinesis. In addition, the endosymbiont is usually found close to the host cell nucleus, presenting different shapes during the protozoan cell cycle. Considering that the endosymbiosis in trypanosomatids is a mutualistic relationship, which resembles organelle acquisition during evolution, these findings establish an excellent model for the understanding of mechanisms related with the establishment of organelles in eukaryotic cells.

## Introduction

Protozoa cell division involves unique morphological changes to accommodate DNA replication with the segregation of single copy and essential organelles. This is the case of trypanosomatids, which present typical morphological features such as the cytoskeleton, composed of subpellicular microtubules, and a flagellum associated with a paraflagellar rod, a trilaminar lattice-like structure that runs alongside the flagellum axoneme. The flagellum, which protrudes from a flagellar pocket, is associated through the basal body, with a single mitochondrion containing a network of circular DNA, called the kinetoplast [1]–[4].

Some monoxenic trypanosomatids, which inhabit an invertebrate host during all its life cycle, present a single endosymbiotic bacterium in their cytoplasm. This bacterium co-evolves through a mutualistic relationship with the host protozoan, constituting a valuable model to understand the symbiotic origin of organelles (reviewed by [5]). When the endosymbiont is present ultrastructural alterations such as, a reduced paraflagellar rod and a looser arrangement of kDNA network, are observed in the host trypanosomatid [6]–[7]. An extensive metabolic exchange maintains both partners together; the endosymbiont contains enzymes that complete the protozoan metabolic pathways [8]–[10], while the symbiotic bacterium may obtain ATP through the activity of host glycosomes, which are organelles that compartmentalize glycolytic enzymes [11]. The symbiont is enclosed by two unit membranes and contains a reduced peptidoglycan layer, which is involved in the bacterium shape maintenance and division [12]. Differently from bacteria, but similar to most mitochondria, it lacks the septum and does not form the FtsZ ring, structures which play essential roles in prokaryote division [13]–[14]. Phylogenetic analyses of ribosomal genes have revealed that the endosymbiont of different trypanosomatid species are similar, being classified in the β division of Proteobacteria, since it is phylogenetically related to bacteria of the *Bordetella* genus [15].

The cell cycle of trypanosomes has been well characterized in *Trypanosoma brucei*, with some studies in *Trypanosoma cruzi*, *Crithidia* and *Leishmania* species [2], [16]–[19]. At the beginning of the cell cycle, trypanosomes present a single flagellum, one kinetoplast and one nucleus. After faithful duplication and segregation of these structures, two new viable cells are produced.

Usually, the cell cycle begins with the maturation of the probasal body and the formation of a new flagellum [18], [20]–[21]. In the procyclic form of *T. brucei*, which replicates in the insect midgut, the kinetoplast S phase initiates immediately before the beginning of the nuclear S phase [22], whereas in *Crithidia*, *Leishmania* and *T. cruzi*, DNA synthesis in the nucleus starts before DNA synthesis of the kinetoplast [16]–[18]. However, in both cases, the kinetoplast S phase finishes before entry into the nuclear G2 phase. The kinetoplast also divides and segregates before the nuclear division [18], [20], [22]. As the cell cycle proceeds, during the nuclear G2 phase basal bodies separate in a microtubule and centrin mediated process, promoting the kinetoplast and Golgi segregation [23]–[24]. In all trypanosomatids, the nuclear chromosomal segregation takes place during a closed mitosis, with formation of an intranuclear spindle without disruption of the nuclear envelope [25]. Later on, the cytokinesis initiates at the anterior end of the protozoan and continues with the ingression of a cleavage furrow along the longitudinal axis of the dividing trypanosome, passing between the two flagella to form daughter cells [20], [26].

The cell division cycle in endosymbiont-bearing trypanosomatids has still not been explored. Previous studies have reported that the symbiotic bacterium divides in synchrony with the host protozoan structures, in such a way that each daughter cell carries only one endosymbiont [5], [14]. Although synchronous, the exact time by which the symbiont divides is unknown. Therefore, in the present work, we describe the morphological events that occur during *Crithidia deanei* cell cycle, in particular the chronological division of the symbiont, relative to the other trypanosomatid host structures, such as the basal body, the kinetoplast, the flagellum and the nucleus. This unique system provides an interesting model to understand the relationship between cell cycle and organelle division processes.

## Methods

### Protozoa growth

*Crithidia deanei* was growth at 28°C in Warren's culture medium [27] supplemented with 10% fetal calf serum. When the culture reached 1×10^8^ cells/ml, it was used for experimental assays. New cultures were obtained after inoculation of 10% of an old culture maintained at 4°C The *C. deanei* generation time is equal to 6 h.

### Transmission Electron Microscopy

For routine transmission electron microscopy, protozoa were fixed for 1 h in 2.5% glutaraldehyde, diluted in 0.1 M cacodylate buffer pH 7.2. Then, cells were washed twice in the same buffer and post-fixed in 1% OsO_4_, 0.8% KFe(CN)_6_, 5 mM CaCl_2_ diluted in 0.1 M cacodylate buffer. Later, cells were washed, dehydrated in a graded series of acetone solutions and embedded in Epon. Ultrathin sections were stained with uranyl acetate and lead citrate before observation in a Zeiss 900 transmission electron microscope.

### FIB–SEM tomography

A FIB–SEM microscope is a scanning electron microscope (SEM) combined with a focused ion beam (FIB) in such a manner that both beams coincide at their focal points. This technique involves the sample serial sectioning with the ion beam, thus generating new block faces, which are then imaged at high resolution with the electron beam. FIB-based tomography is a powerful method for investigating three-dimensional (3D) structure of biological and geological materials, as well as ceramic samples [28].

For observation in FIB-SEM, resin embedded samples were mounted on a support stub for SEM (FEI Quanta 3D DualBeam instrument, FEI Company). A 3 nm layer of platinum was deposited onto the sample, with a sputter coated. Images of the samples were taken with the ion beam at 30 kV acceleration voltage and a beam current of 1000 pA, in the SE imaging mode. The sample was tilted 52° to orientate the surface perpendicular to the Ga^+^ ion beam and a U-shaped trench was cut around the area of interest. For slice and view, the sample was tilted back to 0°, and the block was milled with the ion beam pointing at an angle of 38° to the sample surface, and the dwell time was 0.3 µs. After milling, the sample was ready for slice and view. The slice thickness was 113.5 nm. Images of the cell surface were taken with the electron beam at 5 kV acceleration voltage, beam current at 4000 pA and dwell time 6 µs, in the BSE imaging mode.

### Three-dimensional reconstruction

Models were constructed on computer running MIDAS and IMOD software (Boulder Laboratory, University of Colorado, Boulder, Colorado, USA) [29]. Image stacks were aligned using MIDAS. IMOD was used to stack the aligned images and the structures of interest were traced to provide a 3D representation. Using the IMODmesh feature of IMOD, the contours of each object were joined to form a 3D model. Movies of these models rotating in space were made using Quick Time software.

### Antibodies

The following antibodies were used in this work: (i) polyclonal antibody produced against the recombinant FtsZ of the symbiont, which was kindly provided by Dr. Stenio Fragoso and homogeneously labels the endosymbiont matrix [14], (ii) the MAb (TAT1), which was kindly supplied by Dr. Keith Gull, and recognizes α-tubulin, thus labeling the trypanosomatid cell body and the flagellar axoneme [30] and (iii) an anti-γ tubulin antibody which was kindly provided Dr. Michel Bornens [31] and was used for basal body labeling.

### Immunofluorescence assays

Asynchronous and exponentially growing protozoa were washed in phosphate buffer saline, pH 7.0 (PBS) and fixed by incubation with 4% freshly prepared formaldehyde in PBS for 30 min. Then, cells were deposited on poly-L-lysine-treated microscope slides and permeabilized with 1% NP-40 in PBS for 40 min. The slides were incubated in blocking solution containing 1.5% BSA, 0.5% teleostean gelatin, 0.02% Tween 20 in PBS, and were then incubated for 1 h with the following antibodies diluted in blocking solution: anti-symbiont FtsZ (1∶50), MAb TAT1 (1∶5) and anti-γ tubulin (1∶50). It is worth mentioning that our previous work showed that differently from most bacteria, the symbiont of trypanosomatids does not form a Z ring, a structure involved in bacterial cell division. Thus, the use of anti-FtsZ antibody promotes a homogeneous labeling of the endosymbiont matrix (Motta et al., 2004). After incubation with the primary antibody, protozoa were washed and incubated for 45 min with Alexa 488-conjugated anti-mouse IgG (to FtsZ), Alexa 456 conjugated anti-mouse IgG (to α-tubulin) or Alexa 456 conjugated anti-rabbit IgG (to γ tubulin) diluted (1∶200) in blocking solution. Later, cells were incubated with 4′,6-diamidino-2-phenylindole (DAPI, from Molecular Probes) for 5 min. The pre-immune serum or samples incubated without the primary antibody were used as a control. The slides were mounted in N-propyl gallate and serial image stacks (0.2-µm Z-increment) were collected at 100x (oil immersion 1.4 NA) on a motorized Olympus BX microscope equipped with a differential interference contrast optics, and a Orca R^2^ (Hamamatsu, Japan). All images were collected with Cell∧M software (Olympus), and fluorescence images were deconvolved by using blind deconvolution with the AutoQuant 2.1 software (Media Cybernetics). Alternatively, samples were visualized with confocal laser scanning microscope (Zeiss LSM510 META). Projection of the Z axis and the 3D reconstructions were obtained and processed using the Z-stack systems of the microscope software.

### Cell cycle analysis

The period of the cell cycle that a cell takes with each form of the symbiont (rod shaped, one dividing symbiont or two symbionts) were calculated based on DAPI staining and labeling of the symbiotic bacterium with anti-FtsZ antibody, using the Willians analysis [32]:x = ln (1– y/2)/– α,where x is the cumulative time within the cycle required to reach the end of the period in question, y is the cumulative % of cells up to and including the stage in question (expressed as a fraction of one unit) and α is the specific growth rate. The description of events and timings in the *C. deanei* cell cycle were based on counts of 1000 cells.

## Results

**Figure 1:**
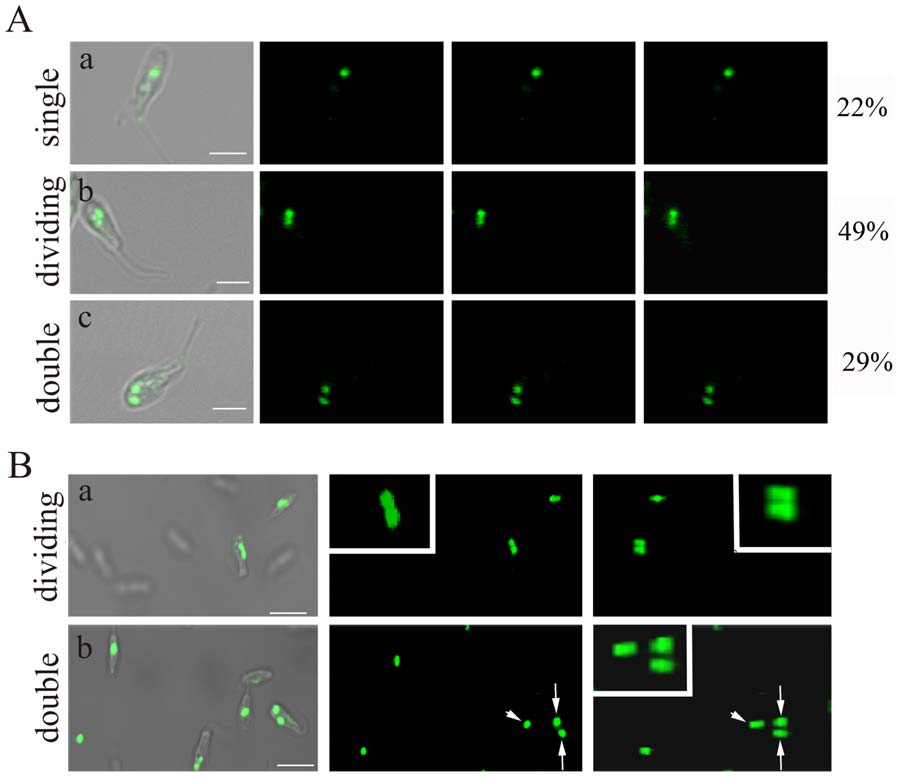
The different symbiont forms during *C. deanei* cell cycle. A non-synchronized cell culture were deposited on slides, previously treated with poly-L-lysine, and incubated with anti-FtsZ to label the symbiont. Cells were observed by confocal microscopy in different sections (A) or by different angles (B). Percentages in A are the proportion of each symbiont pattern (single rod-shape, constricted, double rod-shape) found in an exponential growing *C. deanei* culture. In B the white head arrow shows the cell containing a single rod-shape symbiont and the white arrow shows the cell containing double rod-shape symbiont. Bars = 2.5 µm.

Previous studies have suggested that the symbiotic bacterium divides in synchrony with the host trypanosomatid structures, since each daughter cell carries only one endosymbiont [5], [14]. In order to study the morphological changes that occur with the symbiont during the *C. deanei* cell cycle, we first analyzed an asynchronous culture of this protozoan by immunofluorescence assays using an anti-FtsZ antibody, which specifically recognizes the bacterium. Differently from most bacteria; the symbiont of trypanosomatids does not form a Z ring, a structure involved in bacterial cell division. Thus, the anti-FtsZ antibody produces a homogeneous labeling of the endosymbiont matrix (Fig. 1), as previously described [14]. In asynchronous culture of *C. deanei*, 22% of trypanosomatids presented a rod-shaped endosymbiont (Fig. 1A, single), 49% of protozoa contained a single bacterium with a constriction, thus considered a dividing form (Fig. 1A, dividing) and 29% of cells displayed two rod-shaped symbionts (Fig. 1A, double). In order to certificate that the constricted or dividing form is distinct from the already divided symbiont (double), protozoa were observed by confocal microscopy by different angles (Fig. 1B). Results clearly showed that the host protozoan presents endosymbionts with different shapes. After counting the different symbiont formats in the host trypanosomatid and considering the generation time of *C. deanei* (6 h), we were able to calculate the time that the symbiont persists in each shape. Results, based on Williams cell cycle analysis [32], as described in materials and methods section, revealed that the symbiont remains as a single rod-shape bacterium for about 1.0 h, whereas the constricted form persists for about 3 h. After the bacterial division both symbionts are maintained in the host protozoan for about 2 h.

**Figure 2:**
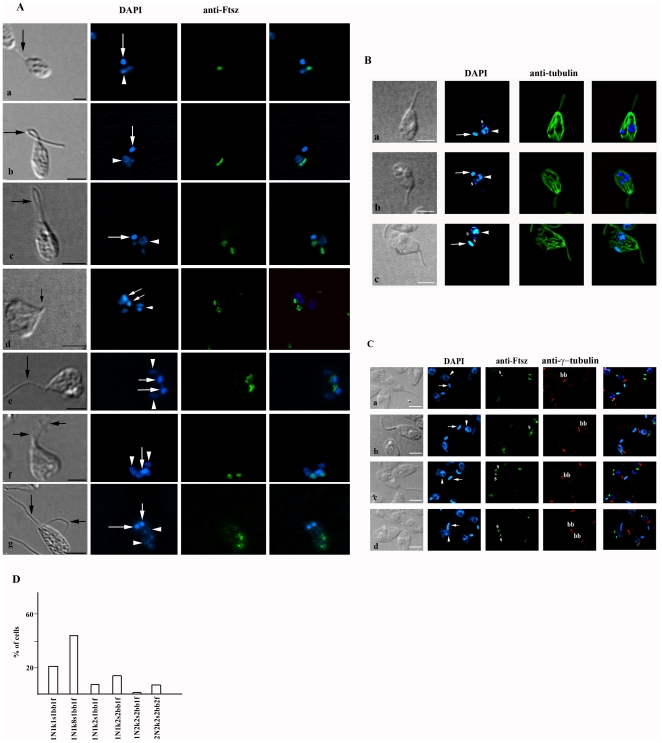
Morphological patterns of exponentially growing *C. deanei*. (A–C) Cells were harvest by centrifugation, washed with PBS and fixed with formaldehyde. Then, cells were deposited on slides, previously treated with poly-L-lysine. In panel A cells were incubated with anti-FtsZ in order to label the symbiont and stained with DAPI (A). This figure (A-a-A-g) shows typical images of each observed cell morphological pattern until cytokinesis. Note that during the cytokinesis the symbiont and the kinetoplast migrates to the posterior end of the protozoan cell body. In panel B cells were incubated with anti-tubulin and stained with DAPI. In panel C cells were incubated with anti-Ftsz, anti-γ-tubulin and stained with DAPI. In A-C: the black arrow indicates the flagellum, while the white arrow shows kinetoplast and the head arrow shows the nucleus. (s) indicates the symbiont and (bb) the basal body. Bars = 2.5 µm. (D) Graph shows the proportion of cells presenting each different morphological pattern concerning the nucleus (N), kinetoplast (k), rod-shape or constricted (∞) forms of the symbiont (s), basal body (bb) and emerged flagellum (f) (n = 1000).

Once established that the *C. deanei* symbiont can present different shapes, we asked how these different bacterial formats were associated to the host protozoan during its cell cycle. So, our next step was to investigate *C. deanei* cell cycle, considering the symbiont shape, number and position, in relation to the nucleus, kinetoplast, basal body and flagellum. Immunofluorescence analysis showed that the symbiotic bacterium can be observed close or far from the host cell nucleus and varied in shape (Fig. 2A). A round shaped symbiont was found in *C. deanei* with single nucleus, kinetoplast and flagellum (Fig. 2A-a). Due to the abundance of this pattern and the presence of single organelles, we interpreted these forms as being in the G1 or S phase of *C. deanei* cell cycle. Another population of cells presents just one nucleus, one kinetoplast, one flagellum and a more elongated and constricted endosymbiont that lies down over the protozoan nucleus (Fig. 2A-b). Less frequently, we observed two bacteria that were symmetrically distributed from each other, considering the nucleus position (Fig. 2A-c). These two symbionts were also present in cells with a single flagellum, nucleus and kinetoplast. This suggests that the symbiont divides before nucleus and kinetoplast segregation, and before the appearance of the new flagellum. After symbiont division, the kinetoplast divides, since we could find cells with two rod shaped symbionts, two kinetoplasts, one nucleus and a single flagellum emerging from the flagellar pocket (Fig. 2A-d and movie S1). The next step on the cell cycle is the nuclear division (Fig. 2A-e). At this cell cycle phase the trypanosomatid presents a “heart-shape”, where both kinetoplasts have a posterior position in relation to the nucleus and are close to rod shaped symbionts. Then the new flagellum emerges from the flagellar pocket (Fig. 2A-f). It is important to note, that at this cell cycle stage, the replicated rod shaped symbionts are located at the posterior end of the host cell, while the segregated kinetoplasts localizes at the anterior part of the trypanosomatid cell body (Fig. 2A-g). It means that the kinetoplast position changes during cytokinesis. At the end of cytokinesis, the new flagellum reaches the size of the old one and the protozoan assumes a more elongated form (Fig. 2A-g). The two daughter cells are formed presenting a single number of structures, including a single rod-shape symbiont that remains in the posterior region of the cell body, while the kinetoplast is observed close to the nucleus, like in paramastigote forms shown in Fig. 2A-a, when a new cell cycle of *C. deanei* begins.

In order to better visualize the flagellum in relation to other cell structures, protozoa were labeled with anti-α-tubulin (Fig. 2B). In such assays, the symbiont was stained with DAPI, considering that the anti-α-tubulin and anti-Ftsz were produced in mouse. Cells presenting one symbiont (Fig. 2B-a), a dividing symbiont (Fig. 2B-b) or even two symbionts (Fig. 2B-c) and just a single flagellum were observed, indicating that the new flagellum only appears after symbiont division. Next, we investigated if the basal body segregation also occurs after symbiont division. Thus, we performed double labeling assays using the anti-Ftsz to recognize the symbiont and the anti-γ-tubulin to observe the basal body (Fig. 2C). Results showed that protozoa harboring a single symbiont (Fig. 2C-a) or a dividing symbiont always contained a single basal body (Fig. 2C-b), whereas protozoa with two symbionts contained one (Fig. 2C-c) or two basal bodies (Fig. 2C-d). Taken together, these data indicate that the basal body segregation only occurs after symbiont division. The images also revealed cells with two basal bodies and just one kinetoplast (Fig. 2C-d), confirming that the basal body segregation occurs before the kinetoplast division. Interestingly, the basal body position is not coincident with that of the bacterium during *C. deanei* cell cycle (Fig. 2C a-d).

**Figure 3:**
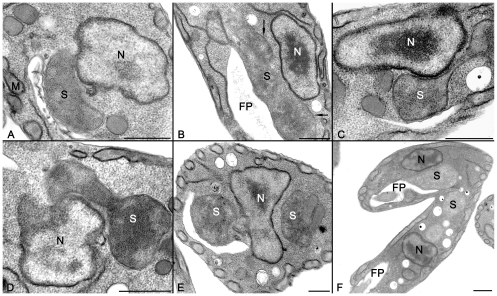
There is a close association between the endosymbiont and the host protozoan nucleus. The symbiotic bacterium was observed as a rod-shape (Fig. 1A and 1B), or forming a constricted structure, which lies down over the nucleus (Fig. 1C) before division (Fig. 1D). Note the symmetrical distribution of the symbionts in relation to the nucleus (Fig. 1E), probably allowing the distribution of equal number of bacteria per protozoan during the cytokinesis (Fig. 1F). FP – flagellar pocket, N – nucleus, S – symbiont. Bars are 0.5 µm.

The quantitative analysis of cellular patterns of *C. deanei* is showed in Fig. 2D. In asynchronous cultures, 22% of cells presented one nucleus, one kinetoplast, one flagellum, one basal body and one symbiont (1N1k1s1bb1f). Most *C. deanei* (49%) presented one nucleus, one kinetoplast, one flagellum, one basal body and one constricted symbiont (1N1k8s1bb1f). Just 7% presented two symbionts and a single copy of other structures (1N1k2s1bb1f). In 14% of protozoa the symbiont and the basal body were found duplicated while nucleus, kinetoplast and flagellum were observed as single structures (1N1k2s2bb1f). Cells in mitosis (1N2k2s2bb1f), or in cytokinesis (2N2k2s2bb2f), represented 1% and 7% of the total cell number, respectively. These numbers further confirm that the endosymbiont is the first structure that divides in *C. deanei.* In order to better investigate the physical relation between the nucleus and the endosymbiont, we performed ultrastructural analysis by transmission electron microscopy (TEM). The rod-shaped endosymbiont can be found far from the nucleus (Fig. 3A), but as the bacterium becomes more elongated it is observed closer to this structure (Fig. 3B). Interestingly, during its division process the symbiont presents a constricted shape that lies down over the nucleus (Fig. 3C) and embraces this structure (Fig. 3D and 3E). This bacterium position probably assures that each new daughter cell will contain just a single symbiont (Fig. 3F).

**Figure 4:**
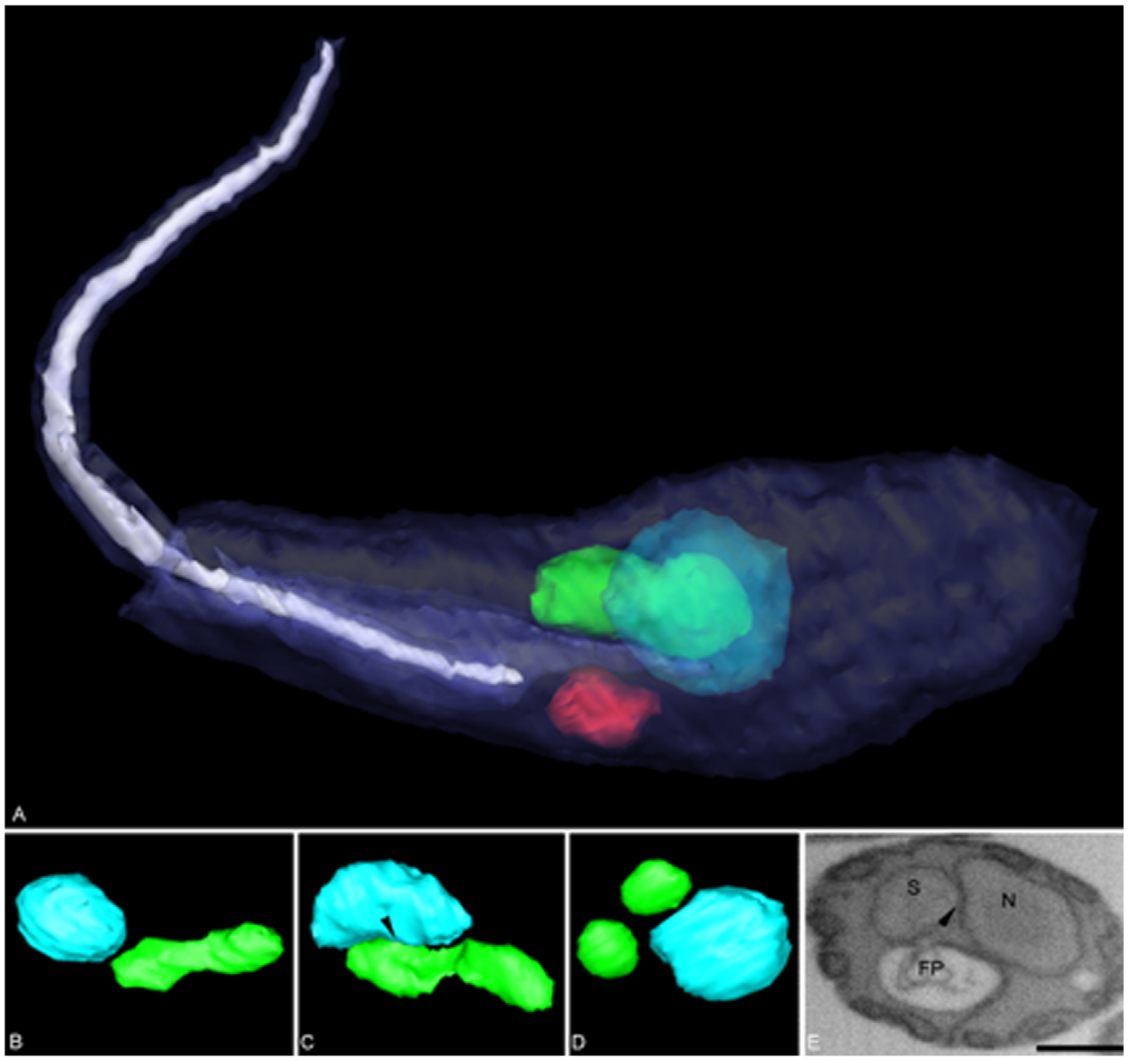
Three-dimensional reconstruction of *Crithidia deanei*. Three-dimensional reconstruction was obtained by focused ion beam associated to the scanning electron microscope (FIB-SEM) tomography. Note the close association between the endosymbiont (green color) and the nucleus (light blue) in figures 2A and C. The protozoan cell membranes are in dark blue and the flagellum in lilac color. The endosymbiont changes its format during the *C. deanei* cell cycle from a rod-shape (Fig. 2B and the supplementary data 2) to a more constricted or dividing form, which is associated to the host cell nucleus (Fig. 2C arrowhead and the supplementary data 3). After division both bacteria are simetrically distributed in relation to the nucleus (Fig. 2A). Thick sections used in the trypanosomatid 3D reconstruction showed an electron dense area, in points where the symbiont is closely associated to the nuclear surface (Fig. 2E-arrowhead). Bar = 0.5 µm.

TEM images of *C. deanei* profiles suggested that the symbiont division is dependent on a close association with the host cell nucleus. However, the random visualization of such phenomenon in thin sections could lead to misinterpretation. Thus, we performed 3D reconstructions of *C. deanei*, by using the FIB–SEM microscope (Fig. 4A). The obtained results generated cellular models showing that the symbiont presents a rod-shape (Fig. 4B and movie S2) or a dumb-bell format, with the handle associated with the *C. deanei* nucleus (Fig. 4C – arrowhead and movie S3). After division, both bacteria present a symmetrical distribution considering the nuclear position (Fig. 4D). Profiles of the protozoa in the surface of the block (oriented in the Z stack), as used for the 3D reconstruction, revealed a more electrondense region in points where the bacterium touches the nuclear surface (Fig. 4E - arrowhead), supporting the idea that the constrained endosymbiont is physically associated to the host cell nucleus.

## Discussion

**Figure 5:**
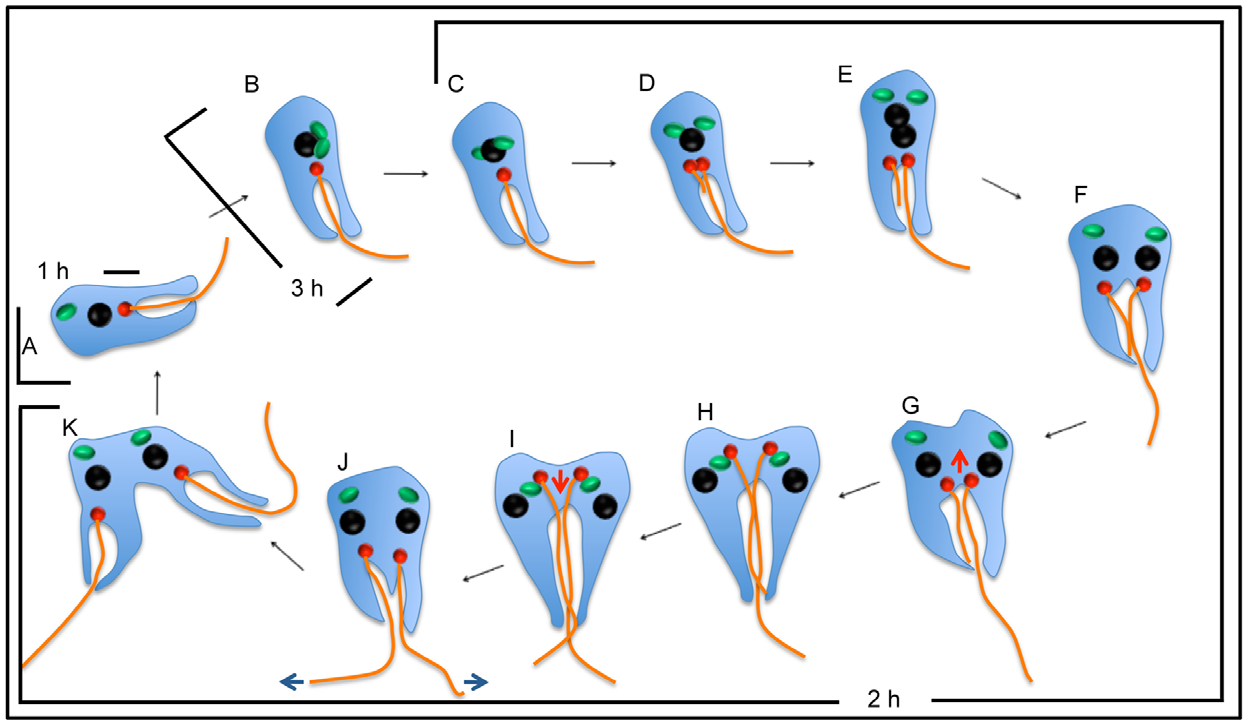
Schematic representation that summarizes the morphological alterations during the *C. deanei* cell cycle. Recently replicated protozoa present a single symbiotic bacterium in rod-shape format (A), the endosymbiont elongates and lies down over the host cell nucleus (B). The bacterium is the first structure to divide (C). After the symbiont duplication, the kinetoplast migrates to the posterior end of the host protozoan (arrow) and the new flagellum grows inside the flagellar pocket (D–E). Then, the kinetoplast segregates (F) and the nucleus divides (G). When the cytokinesis begins, the duplicated bacteria are seen in the posterior end of the protozoan, as well as the duplicated kinetoplasts, considering the nuclear position (G). As the cytokinesis advances kinetoplasts return to the anterior cell end (arrows), while the symbiont remains in the posterior part of the cell body (H–I). The new flagellum only emerges from the flagellar pocket at the end of cytokinesis, when the flagellar pocket probably segregates (Fig. 5 H–J). The flagellar beat in opposite directions (arrows) generates a propelling force in a late dividing protozoan (Fig. 5 I). At the end of the division process each daughter cell contains a single copy structure, including the symbiotic bacterium (J). The symbiont remains as a single rod-shape bacterium for 1.0 h (A), whereas the constricted symbiont persists in this format for 3 h (B and C'). After the symbiont division both bacteria are maintained in the host trypanosomatid for 2 h, before the generation of two new daughter cells (C–K).

Here we describe the morphological events taking place in the cell cycle of *C. deanei* containing an endosymbiont. We also provide clear evidence that the endosymbiont divides before the basal body segregation and kinetoplast and nucleus duplication. Figure 5 illustrates these events. Newly replicated protozoa present single structures, which includes a unique symbiotic bacterium (Fig. 5A). As the cell cycle proceeds the endosymbiont elongates and lies down over the host cell nucleus (Fig. 5B). The symbiont is the first structure to divide (Fig. 5C). After the symbiont duplication, the kinetoplast starts the migration to the posterior end of the cell body and the new flagellum grows inside the flagellar pocket (Fig. 5D-E). Then, the kinetoplast segregates (Fig. 5-F) and the nucleus divides (Fig. 5G). It is interesting to point out that when the cytokinesis begins, the duplicated bacteria and both kinetoplasts are located in the posterior region of the trypanosomatid (Fig. 5G). As the cytokinesis advances kinetoplasts returns to the anterior end of the cell, while the symbiont remains at the protozoan posterior end (Fig. 5H-I). In the choanomastigote form, the new flagellum only emerges from the long flagellar pocket at the end of cytokinesis, when the flagellar pocket probably segregates (Fig. 5 H-J). Since each flagellum beats in opposite directions, this could provide in a late dividing protozoan, a propelling force to generate new daughter cells (Fig. 5 J-K).

Based on the percentage of each endosymbiont pattern in an asynchronous culture during the cell cycle (Fig. 1), as well as the sequence that organelles and structures divide (Fig. 2) we could establish the *C. deanei* cell cycle considering different morphological patterns of this protozoan. The symbiont remains as a single rod-shape bacterium for 1 h after *C. deanei* cytokinesis, when each host protozoan presents just one nucleus, one kinetoplast and one flagellum. After that, the symbiont presents its dividing shape for 3 h when the host protozoan usually presents single copy structures. After bacterial division both symbionts are maintained in the host protozoan for 2 h, before the generation of two new daughter cells, when all structures are replicated.

Previous studies of trypanosomatid have shown that cell division proceeds in the sense basal body-flagellum-kinetoplast-nucleus [18], [20], [22], [33]; this was confirmed in the present work with *C. deanei*, with the bacterium division taking place before basal body segregation. Another interesting aspect of our study is that during the protozoan cytokinesis, the bacterium and the kinetoplast migrates to the posterior end of the host cell. Thus, in this phase of the cell cycle this *Crithidia* species is morphologically similar to the proliferative opisthomastigote forms described in the symbiont-harboring trypanosomatid *Herpetomonas roitmani* [34].

The genome sequencing of the *C. deanei* endosymbiont performed by our group (manuscript in preparation), has showed that some genes pertaining to the division and cell wall cluster were lost by the endosymbiont. This explains in part the symbiont inability to divide outside the protozoan and suggests that the trypanosomatid may provide key elements to the bacterium division. In organelles of symbiotic origin, such as the mitochondrion and chloroplasts, proteins of the bacterial division machinery are partially lost. For example, chloroplasts still divide using components derived from prokaryotic ancestors, as the FtsZ ring. In contrast, mitochondria, which are evolutionarily much older than chloroplasts, lost division components of bacterial origin with the exception of those present in primitive eukaryotes, which retained the Z ring. However, in both organelles, fission depends on dividing rings and dynamin-related proteins, which are associated with the outer membrane and face the cytosol [35]–[36]. Interestingly, obligate intracellular bacteria, like those from *Chlamydia* genus, lack the ftsz genes, as well as a detectable peptidoglycan layer [37]–[38].

Notwithstanding, we should consider that in symbiosis the host cell usually controls the number of associated partners in order to guarantee a perfect relationship. The study of co-evolution between a host protozoan and a symbiotic bacterium has been used to better understand the origin of organelles in the eukaryotic cell. Such studies have shown that the number of prokaryotes per host cell varies from dozens to thousands [39]–[41]. What makes the endosymbiosis in trypanosomatids a special model to study cellular evolution is the fact that each protozoan contains only one symbiotic bacterium, indicating that the host imposes tight control over the endosymbiont division.

The mechanisms by which trypanosomatids control the symbiont division and segregation remains to be elucidated. Our observations suggest that the endosymbiont replication is linked to nuclear fission, since the bacterium shows a close association to the host cell nucleus during the entire protozoan cell cycle. Ultrastructural analysis, by transmission electron microscopy and 3D reconstruction, showed that during the division process, the single symbiont becomes elongated and lies down over the nuclear surface, probably to guarantee a symmetrical distribution of bacteria to both daughter cells. In accordance with this idea, it has been shown that in Apicomplexa parasites, the apicoplast replication is associated with the machinery used for nuclear division [42]. In fact, the segregation of this chloroplast-like organelle depends on a tight association with the protozoan cell centrosome and on the presence of a host dynamin-related protein to guarantee that plastid fission is coincident with daughter cell formation and budding [42]–[44]. However, in this work the basal body position was not seen in coincidence with that of the symbiont during all *C. deanei* cell cycle, indicating that this microtubule-organizing structure is not directly involved in the bacterium division and segregation.

In conclusion, this study shows for the first time that in trypanosomatids, the symbiont division is coordinated with other cellular structures, especially the nucleus. Although the molecular mechanisms involved in the construction, division and segregation of the symbiont remain to be identified, the present work establish a valuable model to study cell division and the origin of symbiotic organelles.

## Supporting Information

Movie S1 3D reconstruction of a cell containing one nucleus (light blue), two kinetoplast (dark blue), two symbionts (green) and two basal bodies (red).
(0.33 MB WMV)

Movie S2 Movie of *C. deanei* three-dimensional reconstruction obtained by FIB-SEM. The symbiont (green) is close to the host protozoan nucleus (light blue), but does not touch this organelle.
(0.53 MB MOV)

Movie S3 Movie of *C. deanei* three-dimensional reconstruction obtained by FIB-SEM. The symbiont presents a constricted or dividing format, which is seen associated to the host cell nucleus (light blue).
(0.81 MB MOV)
